# Commentary: Case Report: Congenital hepatic hemangioma with arteriovenous fistula: 2-year multidisciplinary management and outcomes

**DOI:** 10.3389/fped.2025.1634333

**Published:** 2025-07-17

**Authors:** Huaijie Wang, Zhengtuan Guo

**Affiliations:** Department of Pediatric Surgery & Vascular Anomalies, Xi’an International Medical Center Hospital, Xi’an, China

**Keywords:** hepatic congenital hemangioma, arteriovenous fistula, pulmonary hypertension, atrial septal defect, patent ductus arteriosus, Eisenmenger phenomenon, cerebral infarction, transarterial embolization

A Commentary on Case Report: Congenital hepatic hemangioma with arteriovenous fistula: 2-year multidisciplinary management and outcomes By Mao R, Ruan W, Zhu J, Li L, Jiang H and Li Y (2025). Front Pediatr. 13:1511892. doi: 10.3389/fped.2025.1511892

## Introduction

In the article titled “Case Report: Congenital hepatic hemangioma with arteriovenous fistula: 2-year multidisciplinary management and outcomes”, Mao *et al*. reported a neonate with hepatic congenital hemangioma (CH) treated with multidisciplinary managements, including oral propranolol and transarterial embolization (TAE) (1). We have a few comments on the diagnosis and management of hepatic CH, and some causes of encephalomalacia.

## Comments and discussion

1. Hepatic CH is not infantile hemangioma, oral propranolol is incorrect. For medical management of vascular anomalies, beta blockers are *only* used to treat infantile hemangioma in current evidenced literature. Based on medical history and classic imaging findings provided by authors, a typical hepatic CH can be confirmed. The authors appeared to be unfamiliar with certain classic literature in the field (2–6).
2. Causes of severe iatrogenic encephalomalacia

Arteriovenous shunting and pulmonary hypertension are common in congenital hemangiomas. Following transarterial particulate embolization, embolic materials may migrate into the pulmonary arteries. In a pediatric patient with concurrent pulmonary hypertension, atrial septal defect, and patent ductus arteriosus, a right-to-left shunt (Eisenmenger phenomenon) could develop. This may allow embolic materials to bypass the pulmonary circulation and enter the left heart, ultimately leading to cerebral infarction. Therefore, the encephalomalacia is of iatrogenic origin and unrelated to the congenital hemangiomas. These contraindications of embolization can be found in some classic literature studies (7, 8).

Consequently, this is not a unique baby but a common case of hepatic CH, occurring severe iatrogenic sequelae. Therefore, we suggest that this article should be majorly revised or withdrawn.
